# Strengthening health sector capacities through permanent health education: workshops addressing public health emergencies in Brazil

**DOI:** 10.3389/fpubh.2025.1562010

**Published:** 2025-04-14

**Authors:** Taynná Vernalha Rocha Almeida, Danielly Portes Schelle, Paula Orofino Moura Costa, Isabella de Oliveira Campos Miquilin, Caroline Martins José dos Santos, Paola Freitas de Oliveira, Joelma Ferreira Gomes Castro, Leonora Rios de Souza Moreira, Vinicius Chozo Inoue, Edenilo Baltazar Barreira Filho, Márcio Henrique de Oliveira Garcia

**Affiliations:** ^1^General Coordination of Public Health Emergency Preparedness, Department of Public Health Emergencies, Secretariat of Health Surveillance and Environment, Ministry of Health, Brasilia, Brazil; ^2^Public Health Emergency Training Program, General Coordination of Public Health Emergency Preparedness, Department of Public Health Emergencies, Secretariat of Health Surveillance and Environment, Ministry of Health, Brasilia, Brazil; ^3^Technical Area of Surveillance for Emergencies Involving Chemical, Biological, Radiological, and Nuclear Agents, Public Health Emergency Training Program, General Coordination of Public Health Emergency Preparedness, Department of Public Health Emergencies, Secretariat of Health Surveillance and Environment, Ministry of Health, Brasilia, Brazil; ^4^Department of Public Health Emergencies, Secretariat of Health Surveillance and Environment, Ministry of Health, Brasilia, Brazil

**Keywords:** emergency preparedness, public health surveillance, permanent health education, active learning methods, professional development, Brazil

## Abstract

This initiative strengthens Brazil's Unified Health System (SUS) by enhancing preparedness, public health surveillance, and response capacities to public health emergencies across 27 Federative Units (FUs). Employing andragogical active methodologies, 156 action plans were developed, addressing preparedness (34.7%), public health surveillance (31.8%), and response (33.5%). Training in small groups fostered situational analyses, collaborative planning, and practical application of theoretical knowledge, creating a scalable and replicable model for nationwide implementation.

## 1 Introduction

The COVID-19 pandemic and other recent public health emergencies have exposed structural gaps in workforce preparedness, underscoring the urgent need for permanent health education programs that move beyond traditional, episodic training. Strengthening the capacity of the health sector is not only crucial for responding to immediate crises but also for building long-term resilience and adaptability.

This study introduces an innovative educational model that leverages active learning methodologies to enhance training in preparedness, surveillance, and emergency response. Designed to be both scalable and replicable, this model ensures applicability across different public health settings, promoting continuous workforce development. By aligning with global health priorities—such as the International Health Regulations (IHR) and the WHO's framework for health emergency preparedness—this initiative contributes to a more responsive, agile, and well-prepared health workforce. The approach aims to embed a culture of continuous learning and preparedness, enabling health professionals to anticipate, respond to, and mitigate public health threats effectively.

In the context of Brazil's Unified Health System (SUS), the federal level of management launched the “Workshops on Preparedness, Public Health Surveillance, and Response to Public Health Emergencies” initiative. These workshops provide training on emergency management, contingency plans, standard operating procedures, and roles, fostering active participation and collaboration across various sectors (1). Additionally, these workshops involve guided discussions, and the development of practical materials aligned with proposed objectives, enabling longitudinal monitoring of progress and challenges identified.

Findings in the literature confirm these results both among participants and at the institutional level. Evidence suggests such educational activities increase knowledge and confidence in emergency management while identifying both strengths and limitations in current protocols (1).

The workshops utilized active methodologies and evidence-based practices, ensuring scalability and replicability nationwide. Participants engaged in practical activities, developing action plans for scenarios such as infectious disease outbreaks, chemical accidents, and natural disasters.

The project involved a broad range of health professionals and organizations, including primary care workers, epidemiological surveillance teams, and disaster management entities. It also included Strategic Information Centers for Health Surveillance (CIEVS), the National Hospital Epidemiological Surveillance Network (Renaveh), the Public Health Surveillance Program for Disaster-Related Risks (Vigidesastres), and areas of environmental and laboratory surveillance, as well as disaster control. Entities such as Civil Defense, social participation instances like the National Health Council (CNS), and managerial representations like the National Council of Municipal Health Secretariats (CONASEMS) and the National Council of Health Secretariats (CONASS) also participated.

Organized by the Department of Public Health Emergencies of the Secretariat of Health Surveillance and Environment of Brazil's Ministry of Health and supported by the Pan American Health Organization (PAHO), the project also had the participation of the Special Secretariat for Indigenous Health (SESAI) in specific activities.

This study aims to:

- Develop and present an educational model that applies active learning methodologies to enhance health professionals' preparedness, surveillance, and response to public health emergencies.
- Assess the scalability and adaptability of this model across diverse institutional and regional contexts.
- Detail the methodological framework of workshops that integrate theoretical knowledge with hands-on exercises to improve emergency preparedness.
- Evaluate how this model can be embedded in permanent health education programs to address gaps in institutional capacity and workforce training.

## 2 Pedagogical frameworks

This study employs a descriptive and exploratory qualitative research design, focusing on the development and documentation of an educational model that applies active learning methodologies to train health professionals in emergency preparedness, public health surveillance, and response. The chosen approach allows for an in-depth understanding of the model's structure, scalability, and applicability across different institutional and regional contexts.

A qualitative approach was selected as the most suitable method, given that the study does not aim to measure effectiveness through statistical comparisons but rather to analyze and document the model's methodological framework and its potential for replication. Therefore, no systematic review or quantitative analysis was conducted.

Data collection consisted of structured documentation of workshop implementation processes, including facilitators' reports, structured observations, and analysis of action plans developed during training sessions. This information was used to illustrate the model's methodological structure and its adaptability to different institutional and regional contexts. Since the study is not designed to assess effectiveness, no comparative or statistical analyses were conducted. The qualitative data serves as a basis for understanding how the model can be structured and replicated in various settings.

### 2.1 Data collection

Data collection was based on structured documentation of workshop implementation processes, comprising:

- Facilitators' reports, detailing session dynamics, challenges, and participant engagement.
- Structured observations, capturing the interaction between participants, the application of active learning strategies, and the adaptability of the methodology.
- Analysis of action plans developed during training sessions, providing insights into the model's practical application and perceived feasibility.

The collected qualitative data serves as a foundation for understanding how the educational model can be structured, adapted, and replicated in diverse settings, supporting its potential integration into permanent health education programs.

### 2.2 Facilitator training

The development of facilitators was rooted in an andragogical approach, focusing on learning aligned with local needs and experiences. The goal was to develop competencies for assertive decision-making, allowing facilitators to utilize available resources effectively to address identified needs (2, 3). This approach aimed to form autonomous, reflective professionals.

The facilitator training plan considered the specific themes for each facilitator per Federative Unit (FU). An action plan was created collaboratively by all participants and facilitators from each FU.

Pre- and post-workshop alignments were conducted through briefings and debriefings sessions between managers, organizers, and facilitators. The briefings outlined necessary actions, responsibilities, and timelines, helping facilitators prepare, anticipate challenges, and understand their contributions. These sessions also fostered group cohesion and encouraged collaborative strategy development (4).

After each workshop, debriefing sessions played a key role in learning. These sessions were not just reviews of actions, but opportunities to reflect, analyze performance, and identify strengths and areas for improvement. Sharing experiences and providing constructive input during these discussions strengthened team bonds and promoted a culture of continuous improvement, where mistakes were seen as learning opportunities (5, 6).

### 2.3 Methodology selection

Small group work is a core element in implementing active methodologies. This approach facilitates interaction, discussion, and experience exchange, providing a safe and engaging environment for participants, especially during practical activities (7–9). For each session, participants were grouped into subgroups of 8 to 15 members.

**Figure 1:**
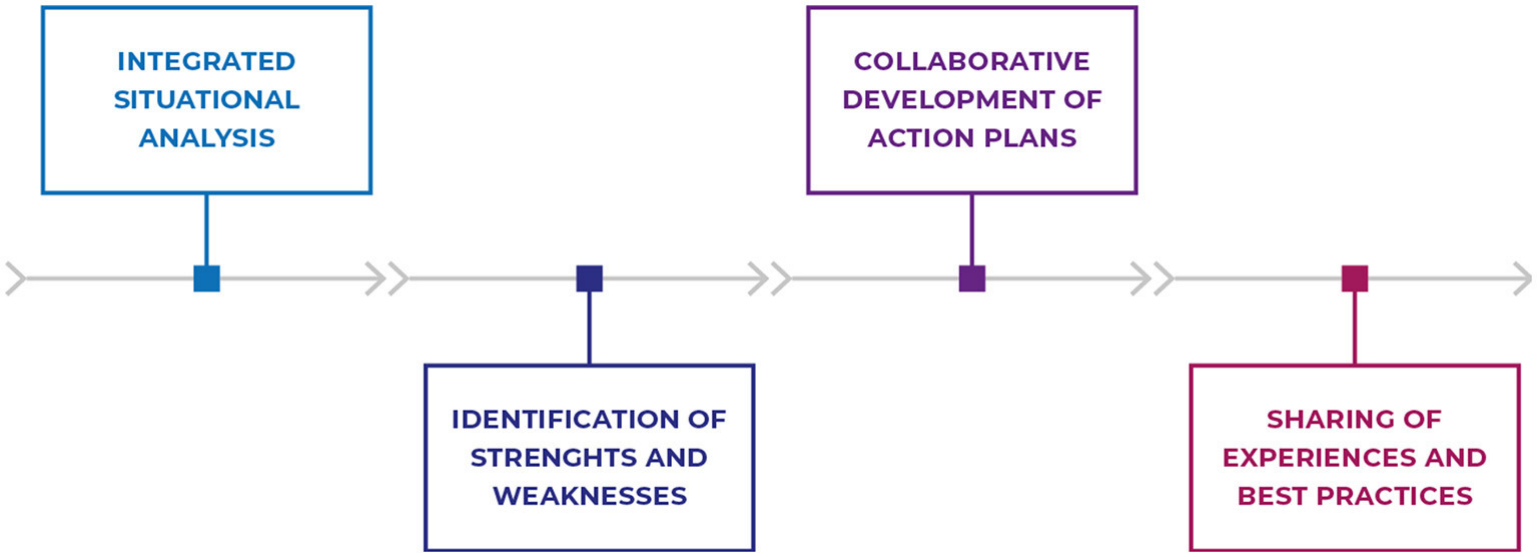
Flowchart illustrating the stages of the methodology applied in workshops on preparedness, public health surveillance, and response to public health emergencies. Source: The authors (2024).

The four stages of the workshops were designed to address specific needs in managing public health emergencies, including preparedness, surveillance, and response. These stages follow structured procedural sequences, fostering understanding, identification, planning, and sharing of solutions to challenges (Figure 1).

## 3 Learning environment

The workshops aim to strengthen professional capacities through interactive group dynamics and practical applications, fostering collaboration among participants to create actionable solutions. For the methodology to be effectively implemented, it is essential to define the roles of each participant and outline the required procedures and structures. This ensures that all involved parties understand their responsibilities and contribute to the workshop's success, as detailed below.

Key roles in the workshop include a methodology advisor, who presents the methodology, facilitates discussions, and supports facilitators during application; Facilitators, who mediate group activities and guide product creation; and a management representative, who represents the institution and reinforces workshop objectives. Additional roles, such as the event focal point, territory focal point, primary reporter, and specific reporter, contribute to the logistical, organizational, and documentation aspects of the event. These roles are summarized in Table 1.

**Table 1:** Roles.

**Role**	**Description**
Methodology advisor	Presents the methodology, leads discussions, and supports facilitators during practical sessions.
Facilitators	Guide group discussions, mediate dynamics, and assist in achieving workshop goals.
Event focal point	Oversees logistics, venue preparation, and participant support.
Territory focal point	Coordinates local participation and ensures alignment with territorial contexts.
Management representative	Represents the institution and emphasizes workshop objectives and follow-ups.
Primary reporter	Documents key discussions, outcomes, and activities, compiling a comprehensive final report.
Specific reporter	Records group discussions and outcomes in detail for dissemination.

Source: The authors (2024).

Procedures and structures necessary for the workshops include preparatory briefings to align roles, select scenarios, and clarify objectives; auditorium setup for plenary sessions with suitable seating and technological support; and a support room for material coordination. Additionally, basic and specific setups ensure all materials are ready before the event, while debriefing sessions provide daily and final evaluations, facilitating feedback and identifying lessons for improvement. These procedures are detailed in Table 2.

**Table 2:** Procedures and structures.

**Procedure/structure**	**Description**
Briefing	Preparatory meeting to align roles, clarify objectives, and select scenario.
Auditorium setup	Preparing the main venue with suitable seating and technological resources.
Support room	Dedicated space for organizing materials and providing logistical support.
Basic and specific setups	Ensuring all materials for group dynamics and activities are ready in advance.
Debriefing sessions	Daily and final evaluations to capture lessons learned and foster continuous improvement.

Source: The authors (2024).

### 3.1 Setup phase

Before the workshop, facilitators participate in a briefing where the general aspects of the event, the methodology to be applied, and the roles of each team member are discussed. During this meeting, hypothetical situations and guiding questions for the activities are also selected. It is recommended to keep a record of these elements for potential reuse in future workshops. This preparatory phase is crucial to ensure all team members understand the workshop dynamics and are equipped to conduct the activities effectively.

### 3.2 Kickoff and methodology briefing

The workshop begins with an opening session featuring local authorities who represent the highest positions of the entities involved. This formal opening highlights the importance of the event and its alignment with public health policies.

Following the opening, a representative from the proposing entity delivers a presentation to outline the workshop's objectives and the significance of the planned activities. Next, a state surveillance team representative presents the local context, emphasizing specific challenges in managing public health emergencies. It is recommended that a report be prepared to document these presentations.

Before the workshop's activities commence, the methodology advisor provides an overview of the methodology, detailing its stages and specific objectives. This briefing ensures that all participants understand the dynamics and are ready to engage. With these initial presentations completed, the workshop transitions into the integrated situational analysis process, marking the official start of activities.

### 3.3 Stage 1: integrated situational analysis

This stage is designed to enhance participants' understanding of the key work processes in managing public health emergencies—namely Preparedness, Surveillance, and Response—through the analysis of hypothetical situations, using guiding questions. Participants are divided into teams, each with a designated facilitator.

Each team must appoint a rapporteur and a spokesperson. The rapporteur is responsible for organizing the team's proposed ideas and recording the relevant information discussed, with a focus on developing the action plan. The spokesperson will prepare the presentation and concisely communicate the team's findings.

Following team introductions, it is recommended to read and analyze the hypothetical situation selected during the facilitator's briefing. Participants should be informed that the scenario serves as a starting point for broader reflection on the applicability of work processes in their respective professional contexts. Participants should also consider other possible situations, including real cases from the territory, thereby expanding the group's perspective.

To encourage the exchange of ideas, a brainstorming session will be conducted. All participants are invited to share their thoughts, emphasizing the realities of their local contexts. This activity encourages diverse insights while ensuring alignment with the team's collective experiences.

During the discussion, previously selected questions should be introduced both directly and indirectly, following the logical order of the work processes: preparedness, surveillance, and response. Participants should be encouraged to document their conclusions and reflections— either by taking notes on paper or using electronic devices—in addition to the rapporteur's role. The accumulation of these records will aid in crafting a well-organized action plan.

The facilitator must ensure the discussion stays focused on the objectives, steering clear of topic deviations. The facilitator should remain attentive to any clarification needs, providing necessary support, as participants are in the process of integrating new concepts (10).

Additional recommendations for the methodology include:

- Create an inclusive environment where all members feel encouraged to actively participate, ensuring they feel safe to share their ideas.
- Prioritize collaboration and alignment with local realities, which are crucial for successful discussions.
- Remind participants of the objectives before the discussion to maintain focus and ensure the work aligns with the intended goals.
- Provide equal opportunities for participation, ensuring all voices are heard.
- Acknowledge and value individual contributions, recognizing diverse perspectives presented by the group.
- Summarize key points periodically to consolidate progress and ensure participants are engaged with the ongoing discussion.

The facilitators must maintain neutrality, avoiding any personal influence on the direction of the discussions. Their role is to foster a collaborative and participatory environment, where all team members are encouraged to contribute constructively.

Additionally, it is vital that participants relate the discussions to the specific realities of their state or municipality. The facilitators should encourage contributions grounded in these local contexts, integrating them into the discussion to enrich the dialogue and ensure the proposed solutions are practical and applicable.

### 3.4 Stage 2: identification of strengths and critical points

The aim of this stage is to assist participants in identifying strengths and critical points within each work process to develop the action plan. Several procedures are recommended for this phase.

Firstly, identify the strengths and critical points within the areas discussed in the work processes of preparedness, public health surveillance, and response. To organize the group, the rapporteur should record the points identified. It is recommended to use sticky notes, markers, and a flipchart. If necessary, the spokesperson can be assigned to expedite the process by recording the strengths and critical points.

During this stage, classic group dynamic techniques, such as those used in design thinking, can be employed (11):

- Brainstorming: This technique is used to identify the main ideas of the team. The facilitator poses a trigger question, and participants succinctly and objectively verbalize their ideas. The facilitator should list the ideas on a board or flipchart.
- Brainwriting: Similar to brainstorming, this technique captures participants' ideas in writing. The facilitator poses a trigger question, and participants write their ideas on sticky notes succinctly and objectively. The facilitator then organizes the notes on a board or flipchart, identifying the main ideas and those that answer the trigger question.

Additionally, the facilitator should ensure that participants understand the importance of identifying both strengths and critical points, as both are essential for developing an effective action plan.

### 3.5 Stage 3: collaborative construction of action plans

The objective of this stage is to engage participants in developing specific action plans for preparedness, public health surveillance, and response to public health emergencies, building upon the points discussed in the previous stages. The team's and the rapporteur's records are crucial for recalling key aspects necessary for the action plan.

The following procedures are recommended to guide this stage:

- Presentation of the pre-established model: The facilitator should introduce the pre-established action plan model briefly, highlighting its structure and purpose. This step ensures participants understand how to organize their ideas effectively.
- Development of the action plan: Participants should collaborate to define tasks, assign responsibilities, set deadlines, and prioritize actions. The facilitator plays a central role in maintaining focus and promoting equitable participation.
- Recording key elements: The rapporteur should consolidate the contributions into a structured format, ensuring that all perspectives are adequately captured. This includes specifying tasks, responsibilities, deadlines, and prioritization criteria.
- Presentation preparation: The group should outline a concise presentation of the action plan, emphasizing key strengths, critical points, and priority actions. The presentation should be aligned with the objectives of the work processes and structured using a simple, predefined template.

During this stage, it is essential to maintain a clear focus on the objectives and foster a collaborative environment. The facilitator's role is pivotal in ensuring inclusivity and guiding discussions toward actionable outcomes that reflect the group's collective input.

### 3.6 Stage 4: sharing experiences and best practices

The purpose of this stage is to facilitate the exchange of experiences and best practices among participants, encouraging discussion about common challenges faced in public health emergencies, as well as the specificities of each territory.

Group presentations should prioritize the use of a predefined model to ensure both conciseness and effectiveness. It is important to allocate a predefined amount of time for each presentation, allowing all groups to share their experiences in an organized manner and within the established schedule.

Facilitating a structured sharing of experiences and best practices fosters a collaborative learning environment, where participants can learn from each other's experiences and develop more effective strategies for managing public health emergencies.

## 4 Results

Workshops were conducted across all 27^1^ Brazilian states (FUs), engaging professionals from 401 municipalities, including all state capitals, with a total of 2,241 participants. During these sessions, participants were divided into groups of 8 to 15 members, each facilitated by one facilitator. To date, 156 action plans have been developed, with contributions from 72 facilitators, some of whom participated in multiple workshops.

Participants were invited from state and regional health departments, municipal health departments, and health districts. Following the workshops, a consolidated report of the action plans for each FU was shared with the respective state health departments. Table 3 presents the total number of participants per workshop and their distribution across FUs, including state capitals and macro-regional areas of activity.

**Table 3:** Number of participants by state (FU), capital, and participants' professional areas.

**State (FU)|capital**	**Number of participants**	**Professional area**		

		**VESP**	**VL|VA|VE**	**AS|other**
Acre (AC)	71	16	46	9
Rio Branco	67 (94%)			
Alagoas (AL)	90	12	57	21
Maceió	67 (74%)			
Amapá (AP)	92	57	25	10
Macapá	64 (70%)			
Amazonas (AM)	67	23	20	24
Manaus	45 (67%)			
Bahia (BA)	72	49	12	9
Salvador	40 (56%)			
Ceará (CE)	88	65	14	9
Fortaleza	61 (69%)			
Distrito Federal (DF)	90	55	27	8
Brasília	64 (71%)			
Espírito Santo (ES)	73	22	40	11
Vitória	39 (54%)			
Goiás (GO)	80	41	24	15
Goiânia	55 (69%)			
Maranhão (MA)	112	72	11	29
São Luís	84 (75%)			
Mato Grosso do Sul (MS)	78	32	31	15
Campo Grande	48 (62%)			
Mato Grosso (MT)	75	34	20	21
Cuiabá	51 (68%)			
Minas Gerais (MG)	95	60	26	9
Belo Horizonte	39 (41%)			
Pernambuco (PE)	73	40	22	11
Recife	52 (71%)			
Piauí (PI)	86	18	44	24
Teresina	61 (71%)			
Rio de Janeiro (RJ)	95	55	16	24
Rio de Janeiro	56 (59%)			
Rio Grande do Norte (RN)	92	78	12	12
Natal	54 (59%)			
Rondônia (RO)	89	23	42	24
Porto Velho	64 (72%)			
Roraima (RR)	89	42	36	11
Boa vista	76 (85%)			
Rio Grande do Sul (RS)	73	34	15	24
Porto Alegre	50 (70%)			
Paraíba (PB)	90	36	32	22
João Pessoa	70 (78%)			
Paraná (PR)	92	46	26	20
Curitiba	60 (65%)			
Santa Catarina (SC)	92	51	30	11
Florianópolis	34 (37%)			
São Paulo (SP)	103	57	27	19
São Paulo	76 (74%)			
Sergipe (SE)	83	46	27	10
Aracaju	73 (88%)			
Tocantins (TO)	101	58	29	14
Palmas	78 (77%)			
**Total**	**2,241**	**1,122**	711	416

VESP, Public Health Emergencies Surveillance (CIEVS, RENAVEH, Vigidesastres); VL, Laboratory Surveillance; VA, Environmental Surveillance; VE, Epidemiological Surveillance; AS, Health Assistance; Other, Civil Defense, Anvisa, Military, CONASEMS, and CNS.

Source: The authors (2024).

The average representation of professionals working in the capitals was 68%, while 32% were from municipalities, regional health departments, and health districts (complete list of municipalities can be found in Supplementary material). Among them, 50% worked directly in public health emergency surveillance, 31% in other health surveillance sectors, and 19% in health assistance and other areas.

Of the consolidated action plans, 34.7% focused on preparedness, 31.8% on surveillance, and 33.5% on response. A critical aspect was the identification of weaknesses: 55.8% of the highlighted points were critical, while only 44.1% were strengths. Regarding implementation timelines, 44% of activities were planned for completion within 6 months to 1 year, 38.8% within 2–3 years, and 22.1% within 3–5 years. Notably, at the time of the workshops, 56.3% of the planned actions had not yet been initiated, underscoring the need for sustained follow-up and institutional commitment.

At the end of each workshop, participants were encouraged to provide anonymous feedback via a QR code-accessible questionnaire. The survey assessed different aspects of the event, including relevance of topics, logical flow, methodology, and achievement of objectives. A total of 805 participants from 25^2^ FUs responded, with 68.2% rating the workshop as excellent, 28.9% as very good, 2.6% as fair, and only 0.2% as poor—meaning over 95% of respondents evaluated the event positively.

### 4.1 Key findings and long-term impact

The findings of this study reinforce the importance of structured and continuous training in strengthening health workforce capacity for emergency preparedness and response. The workshops facilitated the identification of institutional gaps and critical areas for improvement, particularly in:

- Intersectoral coordination, necessary for more integrated and effective responses.
- Adaptation of training to local needs, ensuring greater relevance and applicability.
- Sustained institutional support, which is essential for long-term impact and the implementation of action plans.

While the study does not directly assess the measurable impact of these workshops on health system performance, the structured documentation of the training process provides valuable insights into workforce engagement, capacity-building efforts, and areas requiring further investment.

The COVID-19 pandemic exposed significant weaknesses in global health systems, highlighting the urgency of scalable and adaptable training models to prepare for future crises. The educational framework presented in this study offers a structured, replicable, and context-sensitive approach to strengthening public health emergency preparedness.

By aligning with global frameworks such as the International Health Regulations (IHR) and the WHO Health Emergency Preparedness framework, this model contributes to a more resilient and responsive health sector, capable of adapting to evolving threats. Its scalability suggests potential for replication beyond Brazil, in other regions facing similar challenges, reinforcing the global imperative for continuous capacity-building in health emergency response.

## 5 Discussion and lessons learned

The findings of this study highlight significant gaps in existing health sector training programs, particularly the need for structured, scalable, and contextually adaptable educational models for emergency preparedness. Permanent health education workshops play a crucial role in addressing these gaps by incorporating active learning methodologies that foster problem-solving, teamwork, and decision-making in crisis scenarios.

While the study does not evaluate the effectiveness of the training in enhancing practical skills, it identifies key challenges in implementation, such as the need for stronger intersectoral coordination, institutional commitment, and resource allocation for long-term capacity-building efforts. Additionally, regional disparities in preparedness levels emphasize the importance of adapting workshop content to local realities to maximize impact.

From a long-term perspective, structured training models like the one presented in this study can contribute to greater institutional resilience by embedding emergency preparedness into continuous professional development programs. Ensuring sustainability and scalability requires integrating these workshops into national and regional health education policies, alongside mechanisms for continuous monitoring and refinement.

In high-income countries, public health emergency training often benefits from advanced technological resources and simulation-based learning. However, in resource-limited settings, scalable and adaptable training models are essential to overcome logistical and infrastructural constraints. This study bridges this gap by proposing a methodology applicable to diverse institutional and regional contexts, ensuring accessibility without compromising quality.

By documenting the structured implementation of this model across Brazil's Federative Units, the study provides insights that can inform the development of similar training initiatives in other regions facing challenges in health workforce preparedness. The findings suggest that active learning-based approaches can enhance capacity building in emergency response, making this model a valuable contribution to global health education strategies.

Active learning methodologies, commonly used in health professional training, are less frequently applied in professional development settings. By actively engaging participants and offering opportunities for the practical application of knowledge, these methodologies better equip professionals to address the complexities of emergency scenarios (12). Despite challenges in shifting academic environments, researchers and working groups have proposed strategies to address these barriers, such as competency-based education, interactive learning methods, curriculum restructuring, and authentic assessment tied to real-world outcomes (8, 13, 14).

In this study, the integration of situational analysis and small-group discussions fostered an immersive learning environment. Participants engaged in real-world problem-solving, applying theoretical knowledge to practical challenges, which deepened their understanding of key principles. Facilitator training further ensured the effective implementation of the methodology.

Active methodologies also offer additional benefits, such as fostering teamwork, enhancing communication, and strengthening collaboration skills (9, 15). The opportunity for professionals to interact and solve challenges collectively was identified as a key advantage, preparing them for coordinated emergency responses. Moreover, promoting workshops with diverse participants—representing various roles and competencies in public health emergency management—enhanced recognition of different responsibilities and improved coordination capacity through practical collaboration exercises (1).

Nevertheless, the successful implementation of active methodologies depends on adequate resources, institutional support, and professionals with both theoretical and practical expertise in these pedagogical approaches. Time, space, and institutional support are essential to ensuring these teaching methods' effectiveness.

An analysis of workshops on preparedness, public health surveillance, and response to public health emergencies revealed key strengths and challenges. A major strength was participants' adherence to the methodology and completion of activities within the designated timeframe, largely due to facilitators' expertise in moderating discussions. Additionally, the heterogeneity of group members—spanning different roles, sectors, and competencies—enriched debates, fostering a comprehensive analysis of the challenges presented.

Workshop recommendations emphasized fostering intersectoral collaboration, implementing robust risk assessment methodologies, strengthening health surveillance systems, and planning preparedness actions. Federal-level support was deemed essential to enhance sectoral integration, disseminate surveillance initiatives, and encourage greater participation in emergency management. Given the lower participation rates of professionals from municipalities and regional health departments, a key suggestion was to decentralize workshops, implementing the methodology at regional levels and encouraging state health departments to lead these efforts.

The discussions provided a holistic perspective on the challenges and opportunities emergency preparedness and response. Based on these reflections, several key recommendations emerge for improving future training initiatives and policy integration:

1. Institutionalizing the training model: To enhance sustainability, this educational model should be incorporated into national and regional public health training programs, establishing a structured framework for continuous implementation.
2. Adapting training to local needs: Given regional disparities in preparedness, future workshops should be tailored to local contexts to ensure relevance and applicability for diverse health professionals.
3. Strengthening intersectoral coordination: Collaboration between health authorities, emergency response agencies, and local governments is essential for a more integrated approach to public health emergency preparedness.
4. Developing monitoring and evaluation mechanisms: Future workshops should incorporate systematic assessments to track learning outcomes and measure institutional impacts over time.

These strategies can support the long-term integration of permanent health education workshops within the health sector, strengthening overall resilience and enhancing emergency response capacity.

## 6 Conclusion

This study presents a structured educational model based on active learning methodologies to train health professionals in preparedness, health surveillance, and response to public health emergencies. Designed to be scalable and adaptable, the model can be integrated into permanent health education programs, enhancing workforce preparedness and institutional resilience.

By documenting workshop implementation across Brazil's Federative Units, the study identifies key structural and operational challenges in public health training, such as intersectoral coordination, localized adaptation, and long-term institutional support. While it does not assess training effectiveness, it establishes a foundation for refining and expanding workforce development initiatives.

A key strength of this model is its emphasis on real-life scenario immersion and group discussions, which deepened participants' understanding of critical concepts and strengthened essential competencies such as teamwork, communication, and collaboration. The engagement of diverse professionals enriched debates and broadened perspectives on the challenges of public health emergency management. The recommendations emerging from these discussions addressed critical issues, including institutional integration, risk assessment methodologies, and the need for a more structured and coordinated surveillance system.

Despite its demonstrated feasibility in different settings, the model's long-term impact on workforce preparedness and institutional resilience remains an open question. The heterogeneity in workshop implementation across locations may have influenced participant engagement and learning outcomes, and the lack of a comparative analysis prevents direct assessment against other training methods. Additionally, observational bias may have played a role, as workshop outcomes were documented based on participant reflections and facilitator reports.

Future research should evaluate the effectiveness of this approach through longitudinal studies, measuring improvements in knowledge retention, decision-making, and emergency response capabilities. Systematic integration into national health education policies, alongside assessments of financial and logistical feasibility, could inform large-scale implementation. Further investigations into contextual factors—such as institutional support, resource availability, and intersectoral coordination—may refine its application.

Given the increasing complexity of public health emergencies, exploring the integration of digital learning tools and hybrid training approaches could enhance this model's reach and sustainability. Technology-enhanced strategies, such as simulations and virtual training modules, could provide continuous capacity-building opportunities for health professionals.

By addressing these gaps, future research can contribute to the development of a globally relevant framework for strengthening health sector capacity in emergency preparedness, ensuring that training efforts remain responsive to evolving public health challenges.

## Data Availability

The original contributions presented in the study are included in the article/Supplementary material. Further inquiries can be directed to the corresponding author.

## Footnotes

^1^It is important to note that in the state of Pará (PA), the methodology used was not identical, and therefore, the data from this state were not included in the analysis.

^2^The states of Pará and Piauí were excluded from the analysis due to technical issues.
