# Supplementary material for: Strengthening health sector capacities through permanent health education: workshops addressing public health emergencies in Brazil
Source: Front Public Health. 2025 Apr 14;13:1562010. doi: 10.3389/fpubh.2025.1562010 (PMC12034649; doi:10.3389/fpubh.2025.1562010)
Supplement: Supplementary file 1 [file Data_Sheet_1.pdf]

Supplementary Material

1 Supplementary Data

Supplementary Data 1. List of States and Municipalities Represented in the Workshops.

State	Number of Municipalities Represented	Representative Municipalities
Acre	5	Acrelândia; Brasiléia; Cruzeiro do Sul; Rio Branco; Sena Madureira.
Alagoas	15	Arapiraca; Barra de São Miguel; Capela; Coruripe; Delmiro Gouveia; Dois Riachos; Girau do Ponciano; Maceió; Mata Grande; Palmeira dos Índios; Penedo; Pilar; Rio Largo; São Miguel dos Campos; União dos Palmares.
Amapá	9	Santana; Macapá; Laranjal do Jari; Ambé; Oiapoque; Tartarugalzinho; Calçoene; Pedra Branca do Amapari; Cutias.
Amazonas	13	Atalaia do Norte; Barreirinha; Humaitá; Ipixuna; Lábrea; Manacapuru; Manaus; Parintins; Presidente Figueiredo; São Gabriel da Cachoeira; Tabatinga; Tefé; Tonantins.
Bahia	15	Alagoinhas; Barreiras; Brumado; Eunápolis; Ilhéus; Itabuna; Itacaré; Jacobina; Jequié; Juazeiro; Salvador; Santo Antônio de Jesus; Sobradinho; Teixeira de Freitas; Vitória da Conquista.
Ceará	17	Boa Viagem; Caucaia; Fortaleza; Irauçuba; Itapipoca; Juazeiro do Norte; Limoeiro do Norte; Maracanaú; Maranguape; Novo Oriente; Paraipaba; Moraújo; Quiterianópolis; Quixadá; Sobral; Tabuleiro do Norte; Tianguá.
Distrito Federal		Brasília; Taguatinga; Guará; Gama; Núcleo Bandeirante; Brazlândia; Ceilândia; Planaltina; Recanto das Emas; Santa Maria; Sobradinho; São Sebastião; Águas Claras; Candangolândia.

State	Number of Municipalities Represented	Representative Municipalities
Espírito Santo	9	Barra de São Francisco; Cariacica; Colatina; Santa Leopoldina; Santa Teresa; São Mateus; Serra; Vila Velha; Vitória.
Goiás	13	Anápolis; Aparecida de Goiânia; Campos Belos; Catalão; Formosa; Goiânia; Itumbiara; Jataí; Piracanjuba; Pirenópolis; Posse; Rio Verde; Uruaçu.
Maranhão	17	Açailândia; Bacabal; Balsas; Barra do Corda; Caxias; Chapadinha; Coroatá; Imperatriz; Lago da Pedra; Monção; Paço do Lumiar; Paulinho Neves; Pinheiro; Pio XII; Santa Inês; São Luis; Timbiras.
Mato Grosso	18	Água Boa; Alta Floresta; Barra do Garças; Cáceres; Canarana; Colíder; Cuiabá; Juína; Paranatinga; Peixoto de Azevedo; Pontes e Lacerda; Porto Alegre do Norte; Primavera do Leste; Rondonópolis; São Félix do Araguaia; Sinop; Tangará da Serra; Várzea Grande.
Mato Grosso do Sul	12	Anastácio; Aquidauana; Campo Grande; Corumbá; Coxim; Dourados; Jardim Naviraí; Nova Andradina; Paranaíba; Ponta Porã; Sidrolândia; Três Lagoas.
Minas Gerais	37	Acrelândia; Barbacena; Belo Horizonte; Betim; Campo Belo; Carangola; Contagem; Coronel Fabriciano; Diamantina; Governador Valadares; Itabira; Ituiutaba; Januária; Juiz de Fora; Lagoa Santa; Leopoldina; Manga; Manhumirim; Montes Claros; Paracatu; Passos; Patos de Minas; Pedra Azul; Pirapora; Ponte Nova; Pouso Alegre; Santa Luzia; São Gotardo; São João del Rei; Sete Lagoas; Teófilo Otoni; Ubá; Uberaba; Uberlândia; Unaí; Varginha; Visconde do Rio Branco.
Paraíba	15	Bayeux; Cabedelo; Cajazeiras; Campina Grande; Catolé do Rocha; Cuité; Guarabira; Itabaiana; João Pessoa; Monteiro; Patos; Piancó; Princesa Isabel; Santa Rita; Sousa.
Paraná	26	Campo Mourão; Cascavel; Colombo; Cornélio Procópio; Curitiba; Curiúva; Floresta; Foz do Iguaçu;

State	Number of Municipalities Represented	Representative Municipalities
		Francisco Beltrão; Irati; Ivaiporã; Jacarezinho; Jandaia do Sul; Londrina; Maringá; Paranaguá; Paranavaí; Pato Branco; Paulo Frontin; Ponta Grossa; Porto União; São José dos Pinhais; Telêmaco Borba; Toledo; Umuarama; União da Vitória.
Pernambuco	15	Afogados da Ingazeira; Arcoverde; Caruaru; Garanhuns; Goiana; Ipojuca; Jaboatão dos Guararapes; Limoeiro; Ouricuri; Palmares; Paulista; Petrolina; Recife; Salgueiro; Serra Talhada.
Piauí	11	Bom Jesus; Monsenhor Gil; Paes Landim; Palmeirais; Parnaíba; Picos; Riacho Frio; São Raimundo Nonato; Sigefredo Pacheco; Teresina; Uruçuí.
Rio de Janeiro	16	Águas Claras; Angra dos Reis; Araruama; Belford Roxo; Campos dos Goytacazes; Duque de Caxias; Itaboraí; Maricá; Niterói; Nova Friburgo; Nova Iguaçu; Rio de Janeiro; São Gonçalo; São João de Meriti; São Pedro da Aldeia; Volta Redonda.
Rio Grande do Norte	12	Açu; Cerro Corá; Ipanguaçu; João Câmara; Macaíba; Mossoró; Natal; Parnamirim; Santa Cruz; Santana do Matos; São Gonçalo do Amarante; São José de Mipibu.
Rio Grande do Sul	17	Alto Feliz; Cachoeira do Sul; Canoas; Esteio; Gravataí; Lavras do Sul; Novo Hamburgo; Passo Fundo; Porto Alegre; Rio Grande; Santa Cruz do Sul; Santa Maria; São Borja; São Paulo das Missões; São Vendelino; Uruguaiana; Viamão.
Rondônia	15	Ariquemes; Buritis; Cacaulândia; Cacoal; Costa Marques; Espigão do Oeste; Guajará-Mirim; Jaru; Ji-Paraná; Monte Negro; Ouro Preto do Oeste; Porto Velho; Rolim de Moura; São Francisco do Guaporé; Vilhena.
Roraima	5	Boa Vista; Bonfim; Caracaraí; Pacaraima; Rorainópolis.

State	Number of Municipalities Represented	Representative Municipalities
Santa Catarina	24	Araranguá; Balneário Camboriú; Blumenau; Campos Novos; Chapecó; Concórdia; Criciúma; Dionísio Cerqueira; Florianópolis; Itajaí; Jaraguá do Sul; Joaçaba; Joinville; Lages; Mafra; Nova Erechim; Palhoça; Penha; Rio do Sul; São José; São Miguel do Oeste; Tubarão; Videira; Xanxerê.
São Paulo	14	Botucatu; Campinas; Diadema; Guarulhos; Mogi das Cruzes; Presidente Prudente; Ribeirão Preto; Santo André; Santos; São Bernardo do Campo; São José do Rio Preto; São José dos Campos; São Paulo; Taboão da Serra.
Sergipe	8	Aracaju; Estância; Lagarto; Nossa Senhora da Glória; Nossa Senhora do Socorro; Pojuca; Propriá; Salgado.
Tocantins	11	Araguaína; Augustinópolis; Dianópolis; Guaraí; Gurupi; Miracema do Tocantins; Oliveira de Fátima; Palmas; Palmeirante; Paraíso do Tocantins; Porto Nacional.
